# Knowledge, beliefs, and concerns about bone health from a systematic review and metasynthesis of qualitative studies

**DOI:** 10.1371/journal.pone.0227765

**Published:** 2020-01-15

**Authors:** Jude des Bordes, Seema Prasad, Greg Pratt, Maria E. Suarez-Almazor, Maria A. Lopez-Olivo

**Affiliations:** 1 Department of General Internal Medicine, The University of Texas MD Anderson Cancer Center, Houston, Texas, United States of America; 2 Department of Gastroenterology Medical Oncology, The University of Texas MD Anderson Cancer Center, Houston, Texas, United States of America; 3 Research Medical Library, The University of Texas MD Anderson Cancer Center, Houston, Texas, United States of America; Assiut University Faculty of Medicine, EGYPT

## Abstract

**Background:**

Patients with low bone density or osteoporosis need information for effective prevention or disease management, respectively. However, patients may not be getting enough information from their primary care providers or other sources. Inadequate disease information leaves patients ill-informed and creates misconceptions and unnecessary concerns about the disease.

**Objective:**

We systematically reviewed and synthesized the available literature to determine patient knowledge, beliefs, and concerns about osteoporosis and identify potential gaps in knowledge.

**Methods:**

A systematic search was conducted for full-text qualitative studies addressing understanding, literacy, and/or perceptions about osteoporosis and its management, using Medline, EMBASE, Web of Science, Cochrane Library, CINAHL, ERIC, PsychINFO, Psyc Behav Sci Collec, and PubMed, from inception through September 2016. Studies were selected by two reviewers, assessed for quality, and themes extracted using the Joanna Briggs Institute data extraction tool. Thematic analysis was used to identify themes and subthemes.

**Results:**

Twenty-five studies with a total of 757 participants (including 105 men) were selected for analysis out of 1031 unique citations. Selected studies were from Australia, Canada, Denmark, Norway, the United Kingdom, and the United States. Four main themes emerged: inadequate knowledge, beliefs and misconceptions, concerns about osteoporosis, and lack of information from health care providers. Participants had inadequate knowledge about osteoporosis and were particularly uninformed about risk factors, causes, treatment, and prevention. Areas of concern for participants included diagnosis, medication side effects, and inadequate information from primary care providers.

**Conclusion:**

Although there was general awareness of osteoporosis, many misconceptions and concerns were evident. Education on bone health needs to reinforce areas of knowledge and address deficits, misconceptions, and concerns.

## Introduction

Osteoporosis is a systemic bone condition characterized by low bone mass and deterioration of the microarchitecture of bone tissue, resulting in increased fragility and an enhanced propensity to fracture [1]. The World Health Organization (WHO) operationally defines osteoporosis as bone mineral density of more than 2.5 standard deviations below the young adult mean value [2].

About 54 million Americans are estimated to have low bone mass and thus have an increased risk of fractures [3]. Osteoporotic fractures have a substantial deleterious impact on quality of life, causing pain, restriction of mobility, and depression; mortality rates after osteoporotic hip or vertebral fractures are increased [3]. Low bone mass is estimated to be responsible for over 1.5 million fractures each year in the United States, resulting in 500,000 hospitalizations and nearly 200,000 nursing home placements, at an estimated cost of about $19 billion [4]. This cost is projected to rise about 50% by 2025 [5].

Prevention is key in the control of osteoporosis and its associated fragility fractures. Successful prevention strategies depend on patients’ knowledge and self-efficacy, which work together to affect healthful behavioral modification. Numerous individual qualitative and mixed-methods studies report on knowledge and attitudes about osteoporosis, therefore, we undertook a systematic review of the existing qualitative literature to synthetize the evidence and identify the gaps in knowledge that must be addressed in educational tools on bone health. We used a metasynthesis approach to gain a deeper understanding and insight into patients’ knowledge, beliefs and concerns.

## Methods

The manuscript was prepared according to the Preferred Reporting Items for Systematic Reviews and Meta-Analyses statement (S1 Table) [6].

### Eligibility criteria

We included all full-text qualitative research papers written in English and addressing understanding, literacy, and/or perceptions about low bone density or osteoporosis and its management. Manuscripts that employed a mixed methods design with a substantial qualitative component addressing the study objectives were also included. We excluded studies in which participants were less than 18 years old or in which issues of bone health literacy, concerns, and/or perceptions were not addressed, as well as studies for which only abstracts were available.

### Information sources and search

A systematic literature search was performed by a medical librarian (G.P.) using search terms related to low bone density and osteoporosis in electronic databases including Medline, EMBASE, Web of Science, Cochrane Library, CINAHL, PschINFO, Psc Behav Sci Collec, and PubMed, from inception to September 2016 (S2 Table).

### Study selection

Two reviewers (J.d.B., S.P.) independently screened the manuscripts using the titles and abstracts to exclude studies that were not qualitative or mixed methods studies or did not address knowledge and attitudes on low bone density or osteoporosis. Disagreements were resolved by consensus. Full-text articles of the selected papers were retrieved and assessed.

### Data collection process and data items

Findings from the included primary studies in the form of themes or conclusions made by the authors were abstracted into the Joanna Briggs Institute (JBI)-QARI extraction tool [7] by one reviewer (S.P.) and crosschecked by another (J.d.B.). We collected general study characteristics (i.e., authors, year of publication, number of participants, eligibility criteria, age, and synthesis methods) and findings from individual studies, including aspects of low bone health literacy, knowledge of osteoporosis, beliefs, misconceptions, and concerns about being diagnosed and/or living with osteoporosis.

### Quality appraisal

Papers that met the inclusion criteria were critically appraised for study quality using the JBI appraisal tool [7] independently by two reviewers (J.d.B., S.P.). This tool assesses studies in 10 domains, evaluating congruity among the variables of philosophical position used in the study, study methodology, study methods, data representation, and results interpretation. In addition, the JBI tool assesses the degree to which the researchers make their biases explicit. It also assesses the relationship between what participants are reported to have said and the conclusions drawn. For each domain, an item is appraised as “Yes” if present, “No” if absent, “Unclear” if uncertain, and “Not Applicable” where it does not apply. Results for each of the assessed domains were summarized.

### Synthesis of results

We used a standard metasynthesis approach as described by JBI [7]. The findings were categorized and synthesized using thematic content analysis into themes and sub-themes by one reviewer (J.d.B.) and verified by a second (M.L.-O.). Significant quotes were then tabulated and analyzed in the context of the type of population included in the studies (i.e., studies including only men compared with studies including only women, studies including only people diagnosed with osteoporosis compared with studies including people without an osteoporosis diagnosis, and studies with participants younger than 55 years compared with studies including those aged 55 years or older).

## Results

### Study selection

**Fig 1:**
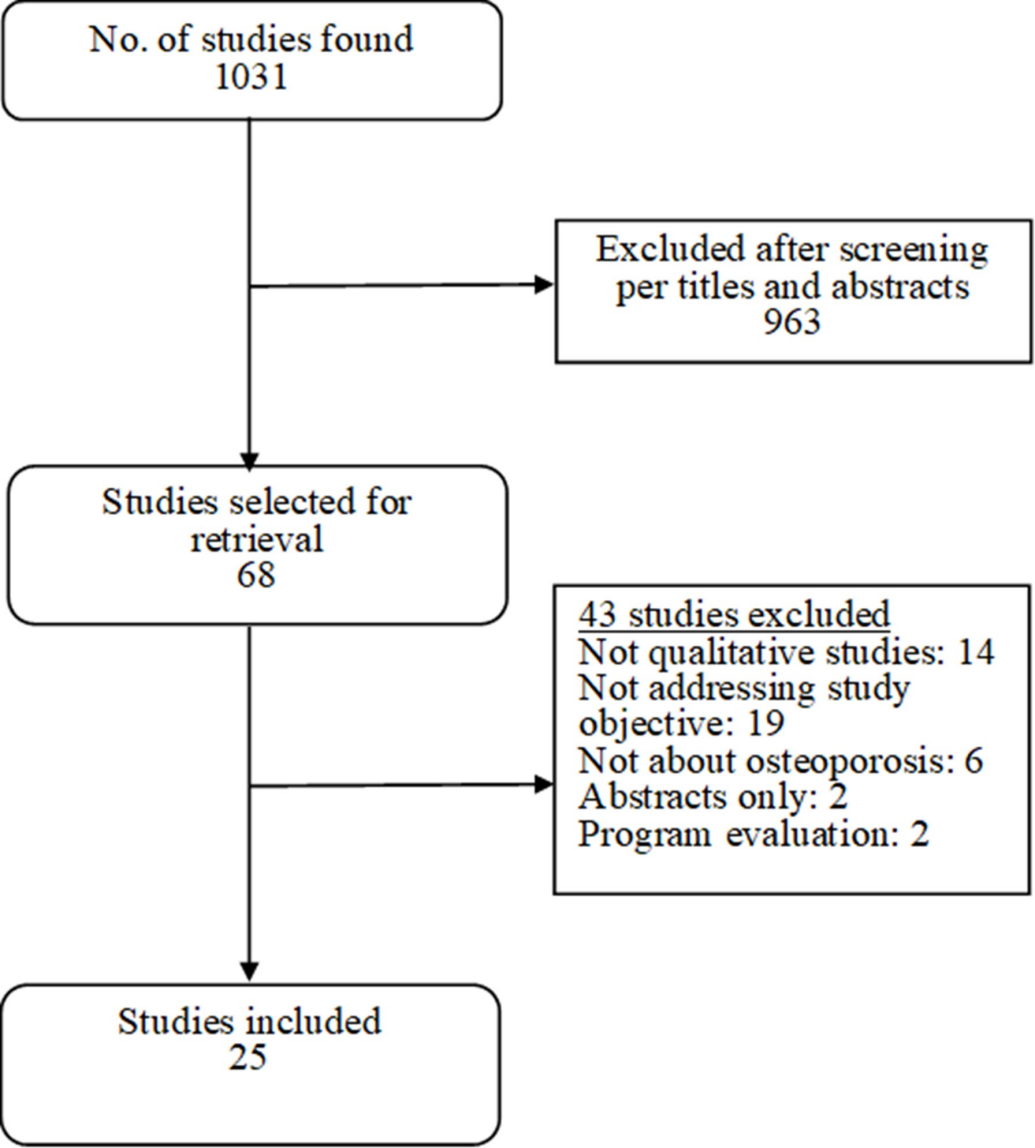
Flow diagram of study selection.

Fig 1 is a flow diagram of the selection of articles for inclusion in the study. A total of 1031 unique citations were identified through the search of online databases. Nine hundred sixty-three studies were excluded after review of titles and abstracts. Sixty-eight studies were selected for retrieval. Of these selected studies, 43 were excluded because they fulfilled one or more of the exclusion criteria (S1 File); 25 studies were ultimately included in the metasynthesis [8–32].

### Study characteristics and participants

A summary of the characteristics of studies included in the metasynthesis is shown in Table 1. The included studies were conducted in Australia, Canada, Denmark, Norway, the United Kingdom, and the United States. Sample size for included studies ranged from 5 to 173. The studies involved a total of 757 participants, 105 (13.9%) of whom were men. Two studies had only male participants [19, 29], and six studies had both male and female participants but the men were in the minority [9, 11, 14, 18, 26, 27]. Three hundred and eighty-nine (51.4%) participants, including 336 women and 53 men, had a history of osteoporosis or low bone density. Thirteen of the studies included participants diagnosed with osteoporosis or low bone density [10, 14–20, 23, 24, 28, 29, 31], and three comprised patients with a history of osteoporosis-related fractures [12, 26, 27]. Three hundred and eigthy-nine (389, [48.6%]) were healthy volunteers or adults in a community living setting with unknown bone health status [8, 9, 11, 13, 21, 22, 25, 30, 32]. The age of participants ranged from 30 to 90 years.

**Table 1:** Characteristics of the studies included in our metasynthesis.

Study	Country	Population	Number of participants	Methods
**Backett-Milburn et al 2000 [8]**	UK	Female employees at a Scottish university, aged 40-55 years	36	In-depth interviews
**Baheiraei et al 2006 [9]**	Australia	Iranian men and women living in Australia, aged 35-77 years	42 men, 131 women	Interviews and focus groups
**Besser et al 2012 [10]**	UK	Women with osteoporosis, aged 69 ± 10.1 years	14	Semi-structured interviews/drawings
**Burgener et al 2005 [11]**	USA	Adult volunteers, aged 65 years or older (mean 75.5 years)	15 (11 women)	Semi-structured interviews
**Edwards et al 2006 [12]**	USA	Postmenopausal women with a history of minimal trauma fractures, aged 61 ± 8 years	29	Prospective studies and focus group (mixed methods)
**Hagy et al 2000 [13]**	USA	White women, aged 30-55 years	39	Focus groups
**Hvas et al** **2005 [32]**	Denmark	White women, aged 52-53 years	17	In-depth interview
**Iversen et al 2011 [14]**	USA	Patients with osteoporosis and receiving osteoporosis medication, aged 65-85 years	32 (30 women)	Focus groups
**Jachna et al 2005 [15]**	USA	Female residents of an assisted living facility who had osteoporosis, aged 71-93 years	5	Semi-structured interviews
**Lau et al 2008 [16]**	Canada	Postmenopausal women receiving osteoporosis medication	37	Focus groups
**Mazor et al 2010 [17]**	USA	Postmenopausal women in a managed care plan who had osteoporosis, aged 73.4 ± 6.2 years	36	In-depth telephone interviews
**Nielsen et al 2011 [19]**	Denmark	Men diagnosed with osteoporosis, aged 51-82 years	16	Focus groups
**Nielsen et al 2013 [18]**	Denmark and UK	Men and women diagnosed with osteoporosis, aged 50-84 years	20 women and 6 men	Semi-structured interviews and participant observation
**Quantock et al 1997 [20]**	UK	Women with osteoporosis, aged 65-76 years	11	Focus groups
**Reventlow et al 2006 [21]**	Denmark	Healthy women, aged 60-61 years	22	Focus groups
**Reventlow et al 2007 [22]**	Denmark	Women, aged 60 years	16	In-depth interviews
**Richardson et al 2002 [23]**	UK	Women with clinical indicators of low bone mineral density, aged 33-81 years	15	In-depth interviews
**Roberto et al 2001 [24]**	USA	Women diagnosed with osteoporosis, aged 53-89 years	21	Focus groups
**Rothmann et al 2014 [25]**	Denmark	Women in a study of the efficacy of a screening program to prevent fracture, aged 65-80 years	31	Focus groups, interviews
**Sale et al 2010 [26]**	Canada	Women and men, aged 47-80 years	18 women 6 men	Focus groups
**Sale et al 2014 [27]**	Canada	Men and women with a history of osteoporosis-related fractures, aged 65-88 years	6 men and 15 women	Semi-structured interviews
**Skolbekken et al 2008 [28]**	Norway	Women invited for bone mineral densitometry, aged 55-75 years	72 women	Focus groups
**Solimeo et al 2011 [29]**	USA	Men with osteoporosis, aged 53-86 years	23	Semi-structured interviews
**Unson et al 2001 [30]**	USA	African-American women, aged 67-86 years	16	Focus groups
**Weston et al 2011 [31]**	UK	Women from a population-based screening trial, aged 68-79 years	10	In-depth interviews

Plus/minus age values represent—means and standard deviations; Ranges are displayed with a dash.

### Quality appraisal

**Fig 2:**
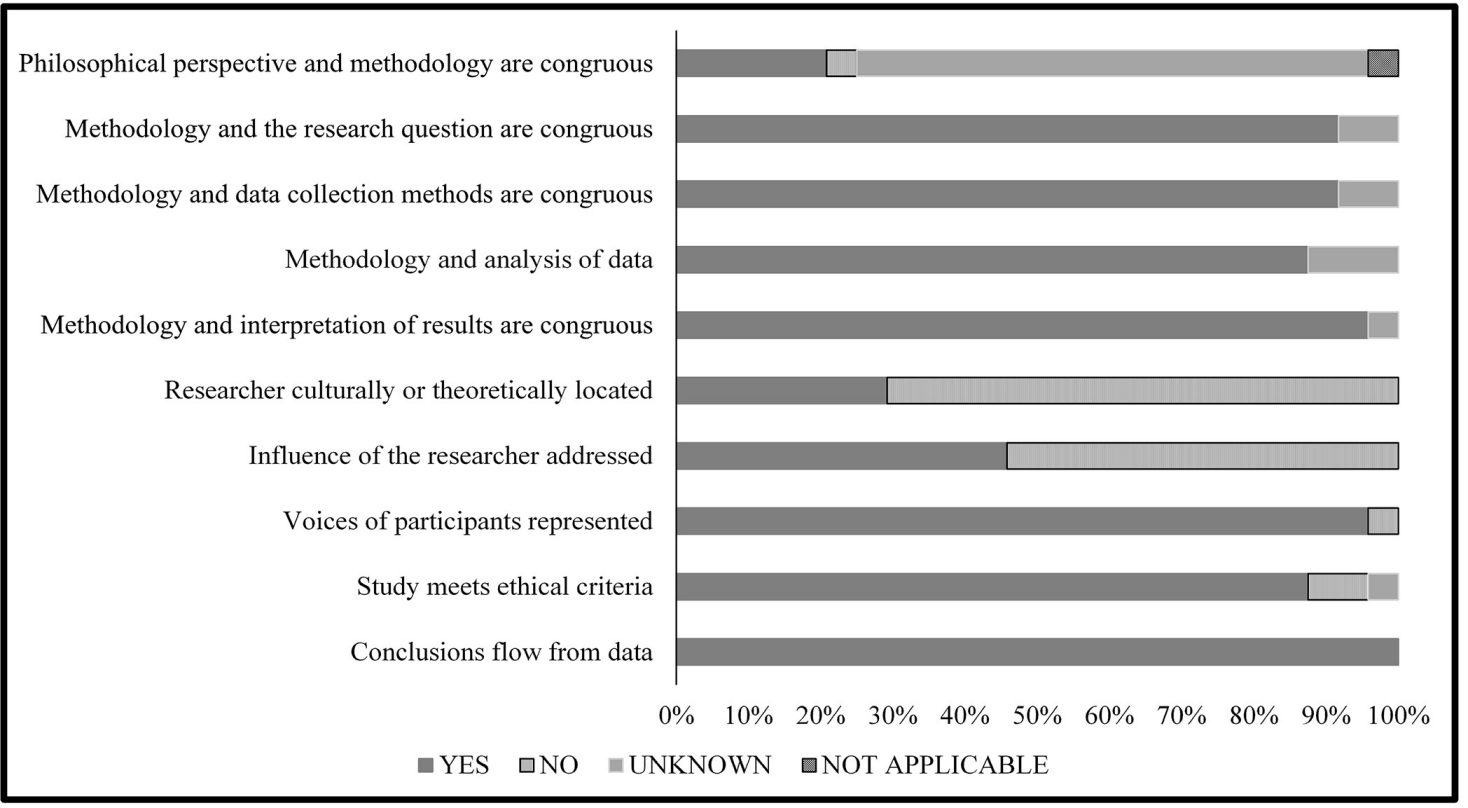
Quality assessment of included studies.

Fig 2 summarizes the final quality assessment of the included studies. Items frequently lacking supporting information to judge quality in the included studies were congruity between the stated philosophical perspective and the research methodology (75.0% of studies), statement locating the researcher culturally or theoretically (66.2%), and influence of the research on the researcher (54.2%). S3 Table shows the individual quality scores for each study. One paper, which was a mixed methods study, was excluded from the quality appraisal [12].

### Synthesis of results

One hundred sixteen findings were abstracted from the included studies and grouped into subthemes. The subthemes were classified into four major themes: inadequate knowledge (i.e., definition and disease course [8–11, 13, 15, 23, 29, 31], risk factors and prevention [9, 11, 13, 20, 21, 26, 27], diagnosis and treatment [11, 16, 17, 26, 27, 29]), beliefs and misconceptions (i.e., etiology, presentation, and perceived seriousness [9, 15, 17, 19, 21, 24–28, 30, 31], perceived personal risk [8, 10–12, 17, 18, 22, 24–26, 28], concerns about osteoporosis (i.e., fear of dependence [9, 10, 14, 20, 24, 27], implications of diagnosis [17, 19, 22, 25, 27], medications [10, 11, 16, 17, 25]), and lack of information from health care providers [9, 10, 14, 20, 28, 29].

#### Inadequate knowledge

Broad knowledge about osteoporosis was common among participants included in the individual studies. However, knowledge of many aspects of the condition was inadequate. Many understood isolated concepts but few had a good understanding of the disease. Knowledge of osteoporosis among participants in 15 included studies was categorized into three subthemes: definition and disease course, risk factors and prevention, and diagnosis and treatment. These subthemes described participants’ understanding of osteoporosis, its risk factors, diagnosis and treatment of the disease, and how it could be prevented. Some participants were simply not interested because they did not see the relevance of osteoporosis to themselves.

**Definition and disease course –** Both men and women knew of osteoporosis. Many participants associated it with weakness of the bones, reduced bone mineral density, and “porousness of bone” or “brittle bone” in elderly people. Some participants in the studies were insecure about their knowledge of osteoporosis and felt that they knew too little about the condition. In some instances, participants confused arthritis with osteoporosis, ascribing joint pain to osteoporosis. Many informants knew that it was a chronic disease but were unsure whether it would advance or improve over their life course.

**Risk factors and prevention –** Some participants in the studies associated osteoporosis with aging, menopause, lack of physical activity, poor nutrition, medications, and family history of osteoporosis. Use of calcium supplements and exercise were known to some participants to be preventive. However, others were uncertain about the types of supplements required, their recommended doses and duration of treatment, or their effectiveness. Some participants understood that low bone mineral density predisposed one to a future fracture.

**Diagnosis and treatment –** Diagnosis of osteoporosis among participants in the studies was usually made when they presented with back or hip pain, through bone health screening, or following a minimal-trauma fracture. Some participants initiated the diagnostic process on their own after obtaining information on the disease from family, friends, or other sources. There was a lack of clarity about the use of the dual-energy x-ray absorptiometry scan and what the results indicated. Some participants were unclear about the types of medications available and how they worked. In some cases, participants familiar with bisphosphonates for the prevention of osteoporosis were uncertain whether the medication might also help with pain. Others sought alternative care such as chiropractic manipulation, physical therapy, and massage for pain after injury.

#### Beliefs and misconceptions

There were misconceptions about many aspects of the disease. Participants’ beliefs and misconceptions of 18 included studies were categorized into two subthemes: etiology, presentation, and perceived seriousness, and perceived personal risk.

**Etiology, presentation, and perceived seriousness –** There was a notion among some participants that osteoporosis was caused by stressful life conditions and unexpressed emotional pain such as sadness and depression. Their image of a person with osteoporosis was one of an old woman with a “bent body and a collapsing back” who had developed the condition through leading a difficult and physically demanding life. Although it was generally expected that collapse of the vertebra or a fragility fracture may be painful, some participants believed osteoporosis was a very painful condition in and of itself. They therefore ascribed pain from many body parts to their osteoporosis. Some participants believed that restriction of physical activities would be beneficial considering their current risk status. There was also a belief that spending too much time thinking about osteoporosis could make the condition worse, and that not much could be done about poor bone health because it is part of the aging process.

In many instances, participants without the disease and those with uncomplicated disease did not see osteoporosis as a serious condition or something that should concern them. This was mainly because it presented with no symptoms or loss of function until one had suffered a complication. Thus, most individuals whose disease was diagnosed at routine screening with no symptoms at that stage did not believe the disease was serious or severe. To some participants, once they were active and could carry out their usual activities, there was no need to worry about the disease. Finally, some participants indicated that osteoporosis is not explicitly life-threatening compared with diseases such as cancer or stroke, reinforcing the belief that osteoporosis is not a serious disease to merit much attention.

**Perceived personal risk –** Many participants did not see themselves as being at risk of osteoporosis even when they acknowledged that osteoporosis and associated fractures could be of great concern. Even some participants who had a history of fragility fractures attributed the cause to reasons other than weak bones, such as the impact of the fall or medical conditions such as glaucoma or poor vision. Some participants believed that they had strong bones and were unlikely to develop osteoporosis. Others saw osteoporosis as no threat to them because no member of their families had ever been diagnosed with the condition. In some cases, respondents saw their risk status as irrelevant because they felt they had no control over their level of risk or risk factors such as aging.

#### Concerns about osteoporosis

Some participants expressed concerns and worries about life after being diagnosed with osteoporosis. Three subthemes were identified under concerns studied in 12 included studies: fear of dependence, implications of diagnosis, and medications.

**Fear of dependence –** Participants who knew about osteoporosis or had vicarious experience of the disease expressed concern about a future of dependence on others due to consequences of severe osteoporosis. They were worried about losing their independence from sustaining multiple fractures, fear of falling, or becoming wheelchair-bound.

**Implications of diagnosis –** For some participants, being diagnosed with osteoporosis evoked concerns and uncertainties. Participants were concerned about their ability to work and socialize. Emotions associated with the diagnosis included shock, anger, and worry. Some were worried about their self-image, such as loss of height or developing a hump at the back. They felt that diagnosis could heighten awareness of their “at risk” state and increase the feeling of vulnerability. They also felt that the diagnostic test, dual-energy x-ray absorptiometry scan, could provide false-positive results and create anxieties. Others felt that the test could reveal other diseases such as cancer. They were also worried that the test could expose them to radiation. However, declining screening could heighten awareness about the body and evoke fear, especially for those who already had pain. Finally, some participants were worried about being diagnosed because they would become “labeled” with a chronic disease and put in a “sick” category, even when the participants were asymptomatic or did not see osteoporosis as a serious disease.

**Medications –** Many expressed concern about medications for osteoporosis, including their side effects, costs, efficacy, and having to take too many medications. Concerns were particularly expressed about media reports linking bisphosphonates to esophageal cancer and having to take some medications on an empty stomach. There were also concerns about the uncertainties of long-term effects of the medications because there were inadequate data on that. Finally, there were concerns about having to take medications for an otherwise asymptomatic disease when many patients were already taking several medications for other chronic diseases. Having to take medications served as a reminder that they were sick.

#### Lack of information from health care providers

This theme was reported in 6 included studies. Participants generally felt that they were not adequately informed about bone health by their health care providers. Some felt that their primary care physicians were not well informed about osteoporosis and they had to find information on their own. Some women felt that male chauvinism was partly to blame for the seemingly inadequate attention paid to the condition because it mainly affects women. Other participants complained that even when they had initiated conversations about their risk with their primary care providers, they were told not to worry about it or that they were not at risk. This contributed to their false sense of not being personally at risk. In one instance, a participant would not accept a relationship between osteoporosis and diet because no health care provider had ever talked about that with her. Table 2 is a summary of the themes with some of their supporting quotations.

**Table 2:** Themes and subthemes with supporting quotations from participants.

Theme	Some supporting quotations
**Inadequate knowledge**	
**Definition and disease course**	“All as I understand, I don’t understand medical terms, but I think it’s an aging of the bones, and the bones can become fragile, and become brittle and they easily break.” (Richardson et al 2002 [23], p. 119)
	“…Actually, I don’t even know…what [osteoporosis] actually does to your body or your bones or whatever it is.” (Burgener et al 2005 [11], p. 675)
	“I mean I think up until a few years ago I mean no one had really heard of osteoporosis. I mean you heard someone’s mother fell and she broke her wrist or something like that, but you never thought it, you just said she was frail or she was older, it always happens” (Backet-Milburn et al 2000 [8], p. 156)
	“I think the crumbling starts, the deterioration of the bones, is most likely to be around the joints where they get wear and tear and that kind of thing. I think it’s to do with little crystals forming…. I presume osteoporosis is something like osteoarthritis.” (Weston et al 2011 [31], p. 1696)
	“…I know that I will never get over it. I am hoping that it can be controlled. I seem to be running downhill with it. I hope that I can better control the pain part of it—control to a point—to forestall severe injury. I am hoping that I’m not going to die with it further in that it is.” (Solimeo et al 2011 [29], p. 537)
	“You don’t get rid of it, do you?” (Besser et al 2012 [10], p. 119)
**Risk factors and prevention**	“Exercise, not just the calcium, but exercise is the key….” (Hagy et al 2000 [13], p. 107)
	“Well, I may be rather stupid, but…I was told to take 1000 mg of calcium and 400 IU of vitamin D and I go into the drugstore and there are all these different types of calcium, some of them less than that…some of them got vitamin D included…. I found out that my multivitamin has got 400 IU of vitamin D, so do I take the calcium with that one? But I got to take another calcium at night, so I’m going insane and my hands are full of little scraps of paper with what different people have told me to take.” (Sale et al 2010 [26], p. 593)
	“I don’t care how much calcium you got…. I don’t care how strong [the bone] is, even an iron can break.” (Burgener et al 2005 [11], p. 675)
	“Certainly I have some osteopenia and my bone density tests which I’ve had over the last four years, it has gone down a small amount each time.” (Sale et al 2014 [27], p. 283)
**Diagnosis and treatment**	“…They (my doctors) found out that I had broken this wrist in 1999 and ankle in 2006 so they suggested that I have the bone density. I don’t understand because it was my wrist and ankle and they checked my back and my hip.” (Sale et al 2010 [26], p. 592)
	“I haven’t seen [osteoporosis literature] other than describing what [osteoporosis] is and that [bisphosphonates] can help it. I think [bisphosphonates] may actually help it in the sense it strengthens your bones. But it doesn’t help with the pain! … It may help me in the future from a future fracture or something. But it doesn’t help the pain here and now. I think it’s a little misleading to people. [laughs] It’s a preventive type of thing, you know. You see it on TV all the time. You know ‘once a month pills’ or ‘once a week pill’ to help prevent a fracture, but, you know, ‘What do you do once you’ve got it?’” (Solimeo et al 2011 [29], p. 537)
**Beliefs and misconceptions**	
**Etiology, presentation, and perceived seriousness**	“If I was in a lot of pain then I’d know that I was in a really bad way, but I’m not, so osteoporosis can’t be that much of a serious problem. Otherwise it’d hurt, and I’d know beyond doubt that it was in my body, um, bones, and causing me lots of trouble.” (Weston et al 2011 [31], p. 1696)
	“I have no problems at all—I had a broken heel and fractures in the back, but now I have no problems…. I am cutting my hedge with arm pads.” (Nielsen et al 2011 [19], p. 169)
	“Like I said, I dance, I jump, I fall down, I get up, I plant, I dig, I plant, I do everything I want. Don’t have a problem. If I do have a problem, I’ll probably do most anything to try to fix it.” (Mazor et al 2010 [17], p. 1003)
	“I feel that [the osteoporosis] is not as life threatening. And certainly at this point is not as serious of a disease, but of course it could change.” (Jachna et al 2005 [15], p. 27)
	“…It isn’t something you die of.” (Rothman et al 2014 [25], p. 191)
	“I’ve got friends who’ve got much worse things wrong with them. I mean, I could have lung cancer or dementia—those poor souls. Now that would be serious and something to worry about. But at least I can take these tablets and be all right. I think I’m lucky that I haven’t got anything much to worry about really.” (Weston et al 2011 [31], p. 1695)
	“…We keep everything inside we’ll get sick and it will affect our bones…it burns our bones from the inside.” (Baheiraei et al 2006 [9], p. 130)
	“I think they simply didn’t know anything about it, our mothers and grandmothers. If they were hunchbacked it was because of the toil…they had very tough lives then our grannies, so they had reason to bend over, to put it that way….” (Skolbekken et al 2008 [28], p. 2566)
	“I guess having children takes…a lot of calcium out of the bones…. And nursing I think does too. That’s why women have more problems with [osteoporosis] than men. If a baby doesn’t get enough calcium through the placenta, then it takes it…out of the mother’s bone.” (Roberto et al 2001 [24], p. 603)
	“I am afraid of diagnosis of the disease as I know I can’t do much about it.” (Baheiraei et al 2006 [9], p. 131)
	“I’m too old I think…I think whatever happened has happened to my body now. I don’t think taking something is going to help it that much.” (Mazor et al 2010 [17], p. 1003)
**Perceived personal risk**	“I think anyone who would have fallen like that would have fractured. It was a hard fall. I am not able to see all that well so I think this is why I fell over.” (Besser et al 2012 [10], p. 119)
	“I broke a bone because I fell. Anyone who falls can break a bone.” (Edwards et al 2006 [12], p. 263)
	“My bones seem to be pretty good. I’ve fallen a few times and I’ve never broken a bone. I’ve never broken anything.” (Mazor et al 2010 [17], p. 1003)
	“I really don’t think I will [develop osteoporosis], because it is really not in my family at all. I’ve never broken a bone in my life.” (Burgener et al 2005 [11], p. 675)
**Concerns about osteoporosis**	
**Fear of dependence**	“ The best thing I can do for other people is to take care of myself so I’m not a burden on them, and I think that’s hard…..it was very hard, I mean extremely hard for someone to give me a bath. That was very hard” Roberto et al 2001[24], Pg. 605
	“This disease is very important because it comes to you when you’re very old, weak and alone…. I am concerned about becoming frail…I don’t want to rely on others.” (Baheiraei et al 2006 [9], p. 130)
**Implications of diagnosis**	“I behave exactly as usual except that when I make my bed for example, or I have to do something or other, then I have to, just like that, ‘oh, your back, now you must just watch out, you know.’ So it’s there at the back of my mind.” (Reventlow et al 2007 [22], p. 163)
	“I see people that have it and the way they’re bent way over due to the back problem and they are in a lot of pain and it’s kind of scary. I visualize myself in 5 years, is that going to be me?” Mazor et al 2010 [17] p.
	“…Now I have back pain, you wonder if you’ve got that crap and whether you were stupid not to get scanned.” (Rothman et al 2014 [25], p. 192)
**Medications**	“I’d read the leaflet about the oesophagus, and a friend…had just died with having cancer of the oesophagus. And it was pretty awful evidently, so I thought, I don’t fancy that…. It probably doesn’t happen to many people, but once you start getting any sort of side effects, you think, oh maybe it’s doing it to me.” (Besser et al 2012 [10], p. 119)
	“I’d rather risk a fall, which could happen tomorrow or it could happen when I’m 80, rather than take something daily that has high risks of side effects.” (Mazor et al 2010 [17], p. 1003)
	“The thing I don’t like about [the medication] is when I get up in the morning, give me that coffee now! And I wait and I’m drinking the water and I’m watching the clock and I’m waiting and I just don’t like that.” (Lau et al 2008 [16], p. 398)
	“It doesn’t affect me at all, I have no symptoms. The only way it affects me is that I have to take medication.” (Besser et al 2012 [10], p. 119)
	“I’ve always been healthy, and I’ve never had to take medication, so having to take medication is like reaffirming you’re getting older and there’s a psychological aspect to that.” (Lau et al 2008 [16], p. 398)
**Lack of information from health care providers**	
	“They (the PCPs) don’t tell you much. It’s printed on your prescription. It’s always printed there and you should be aware of it.” (Iversen et al 2011 [14], p. 75)
	“It’s not been seen as a high status disease, so it’s never been given any priority for that reason. And it’s been a women’s disease, you know. So, the menfolk who sit on the money bags….” (Skolbekken et al 2008 [28], p. 2566-2567)
	“We must have more information about this disease. They give us different options and leave it to us to decide, and when we ask which one is better they tell us it is up to you. How it’s up to us then we don’t know…they don’t care.” (Baheiraei et al 2006 [9], p. 131)
	“Nobody’s ever said to me, if you did this or did that, or ate this or ate that, nobody’s ever given me a diet sheet connected with it, so presumably the powers that be don’t believe diet has anything to do with it because nobody’s ever given me anything to say do this.” (Besser et al 2012 [10], p. 119)

**Fig 3:**
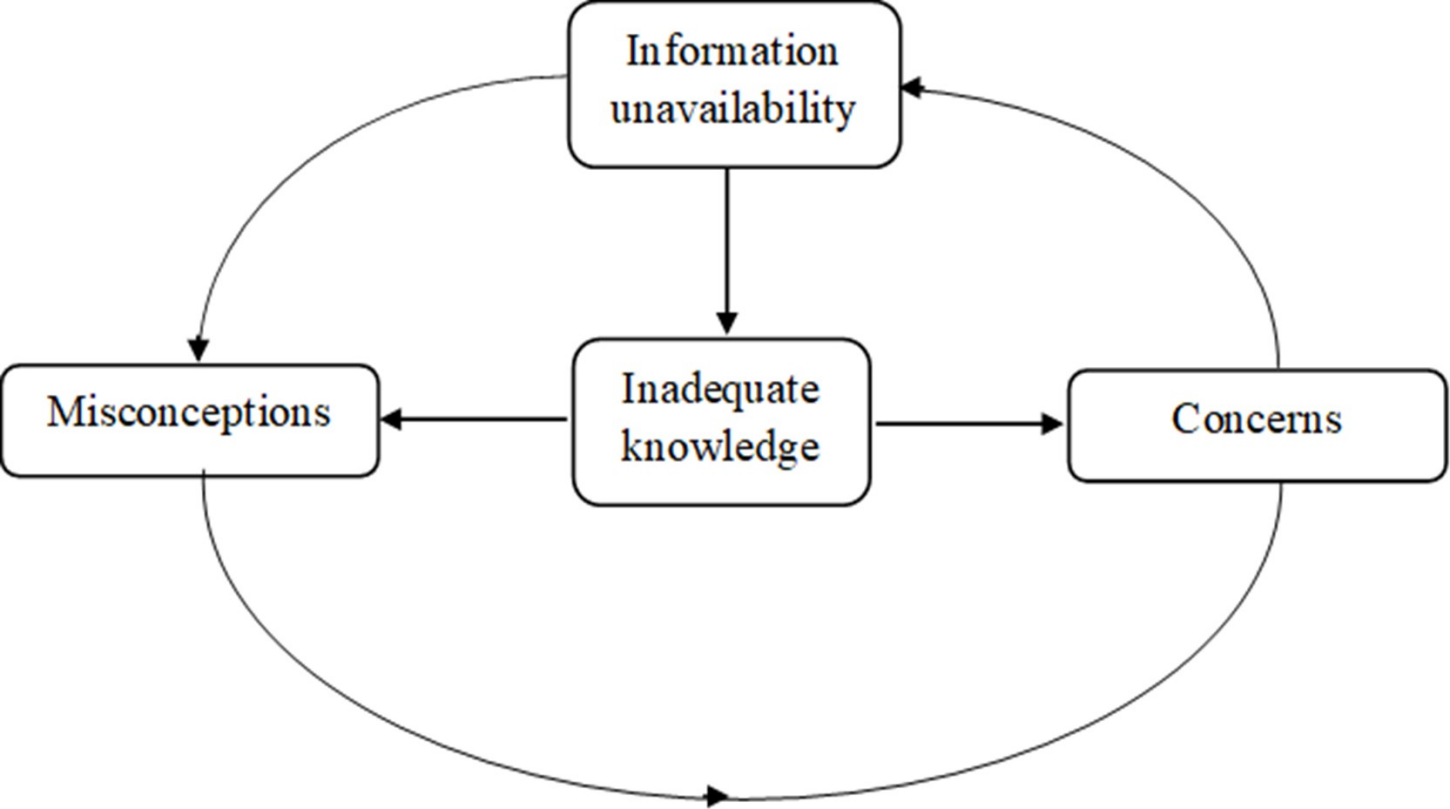
Relationships between the main themes.

Fig 3 summarizes the relationships between the major themes. Information appeared to be unavailable to patients because primary care physicians are not informing them, they do not have access to reliable sources of information, or both. This lack of information leaves patients inadequately informed and breeds misconceptions and unnecessary concerns about the disease. The inadequate information reinforces the misconceptions and heightens patient concerns. Finally, the misconceptions further feed into the concerns.

### Ancillary analyses

We observed differences between certain population subgroups. Men generally had a misconception that osteoporosis was a disease of women and perceived their diagnosis as a weakness. They were not open about their diagnosis and would downplay the symptoms, thus presenting after complications had set in. Some men expressed concern that most educational materials had pictures of women, thus reinforcing their notion that osteoporosis is a women’s disease. Some participants diagnosed with osteoporosis did not believe it could be asymptomatic and ascribed many unrelated symptoms to it. In contrast, those not diagnosed with the disease did not see themselves as potentially at risk. Participants aged 55 years or younger did not express much interest in the condition. Participants older than 55 years were concerned about the association between old age and osteoporosis. Table 3 highlights issues brought up by specific population subgroups.

**Table 3:** Issues brought up by specific population subgroups.

Category	No. of studies	Quotations
**Men**	2 [19, 29]	Misconception: Men perceive osteoporosis as a female disease.
		“What’s wrong with you that you have that? Only women get that…it is embarrassing.” (Solimeo et al 2001 [29], p. 537)
		“…It is like I am walking around telling people that I am suffering from a female disease….” (Nielsen et al 2011 [19], p. 171)
		“You feel kind of odd when you get diagnosed with osteoporosis. You feel like you want to be quiet about it…. You don’t want to tell anybody about it.” (Nielsen et al 2011 [19], p. 171)
**Participants with osteoporosis**	15 [10, 12, 14–20, 23, 24, 26, 27, 29, 31]	Misconception: Some participants did not believe osteoporosis was asymptomatic.
		“I know they say osteoporosis is painless, I can’t really believe that.” (Besser et al 2012 [10], p. 119)
**Participants without osteoporosis**	3 [8, 21, 22]	Misconception: Some participants did not consider themselves at risk or showed little interest.
		“…I mean you’re receptive to what the information is that you want to know and if I’m not into osteoporosis then….” (Backett-Milburn et al 2000 [8], p. 156)
**Aged 55 years or younger**	3 [8, 13, 32]	Misconception: Some participants did not consider themselves at risk and showed little interest.
		“I’m really ill-read on that kind of, you know, my life’s so busy, I hear all these things swanning round the office and I think ‘what’s all this about’ and it hasn’t, you know I’m not into it yet so I’m not really taking time to find out about it….” (Backett-Milburn et al 2000 [8], p. 156)
**Older than 55 years**	11 [11, 14, 15, 17, 20–22, 25, 27, 30, 31]	Concern: Participants were worried about the future.
		“I know old age takes its toll but osteoporosis does hold you back. It makes a difference to the future and the things you can do.” (Quantock et al 1997 [20], p. 47)
		“I am scared of breaking into pieces if I fall, or jump.” (Reventlow et al 2006 [21], p. 323)

## Discussion

We examined qualitative studies that addressed participant knowledge, beliefs, and concerns about bone health. We used a metasynthesis approach to tap into results of many studies that had used qualitative methods to examine participants’ understanding of bone health and its implications. We found that there was widespread awareness but inadequate specific knowledge about osteoporosis. There were many misconceptions and outright false beliefs and concerns among the populations interviewed. We also found that patients at risk of osteoporosis were not receiving adequate information about the condition.

Our finding that inadequate knowledge about osteoporosis is pervasive among studies corroborates the findings of other studies using different methodologies [33–37]. In a review of quantitative surveys on knowledge of osteoporosis, Werner reported serious deficits in knowledge, particularly in the areas of risk factors, consequences of the disease, treatment, and prevention, among various populations, including diagnosed and healthy individuals and even among health care professionals [37]. Although Werner reviewed quantitative studies employing various standardized instruments to assess knowledge, similar findings from our analysis of qualitative studies lend further credence to the observation. A systematic review of quantitative studies of older men’s knowledge about osteoporosis revealed that men had minimal knowledge about the disease process, risk factors, and prevention [33]. Some studies have reported that people who are familiar with the disease either by personal or vicarious experience tend to be more knowledgeable [32, 38]. One study, for example, showed that patients with multiple sclerosis were quite knowledgeable of osteoporosis and its risk factors from interactions with health care providers, although many had not been informed about the association with steroids [39].

Many participants did not see themselves as being at risk of the disease, particularly people older than 55 years who had not been diagnosed. This was found to be partly due to the silent nature of the disease. This was also a common observation in a number of other studies [40–42]. A study in patients with prostate cancer receiving androgen-deprivation therapy, who are at particularly increased risk of osteoporosis, reported a low perceived susceptibility [42]. Our study also showed that many people did not perceive the disease as serious because they did not see it as explicitly life-threatening compared with such diseases as cancer or stroke. Similar findings have been reported in the literature [36, 42, 43]. A study in middle-aged and elderly women showed that although most women (80%) saw osteoporosis as a concern, few (15%) thought it was serious [36]. Younger people did not show concern about the disease probably because it is seen mainly in the elderly.

Patients’ knowledge about a condition can be influenced by a number of factors including education level, ethnicity, language barriers, and socioeconomic status, among others [44]. In addition, our findings of inadequate knowledge, misconceptions, and even concerns about the disease could be attributed in part to lack of information from health care providers. Participants felt that their primary care providers did not adequately apprise them of their osteoporosis risks and educate them on prevention. Similar findings have been reported in other studies [45, 46]. In some studies, patients cite their main source of information on bone health as family and friends, newspapers, television, and the internet [36, 46]. In one such study, physicians ranked fifth as a source of information on osteoporosis [46]. In some instances, patients who found information on their own and raised it with their physicians were told it was not a problem [39]. In contrast, one study showed that physicians were by far the major source of information on bone health, with relatives and electronic media in distant second and third, respectively [47]. Information from mass media or from friends and family may not be reliable and may be a potential source of misinformation, although some credible sites exist on the internet.

Although our study reflected the views of many population groups, including women and men, those with and without osteoporosis, young and old, and participants in various contexts, it had some limitations. Because our study was a qualitative metasynthesis, we analyzed themes and findings from many studies with varied qualitative approaches, contexts, and study quality. Thus our findings may not apply in all contexts. Our systematic search for literature in the databases was up through 2016 and we could have potentially missed some recent studies. However, we did not think further searches would yield any novel findings because we observed a saturation of themes. Finally, as with any systematic review of the literature we are constraint to the data reported in the individual studies. Further analysis to provide detailed information on specific populations such as risk lever (low risk versus moderate risk with known low bone density versus high risk with prevalent osteoporosis fractures), by type of setting (primary versus hospital setting), or minority groups could not be performed.

## Conclusion

Although there was general awareness of osteoporosis, many misconceptions and concerns were evident. Education on bone health needs to reinforce areas of knowledge and address deficits, misconceptions, and concerns using a multi-level approach involving both providers and health information consumers. Primary care providers need to be educated on osteoporosis and its ramifications so they can play a leading role in educating patients on prevention, screening, and treatment. Resources for self-education also need to be available to individuals and the general public. Since many people already look to the internet for health information, making credible resources available online could go a long way in addressing the information gaps.

## Supporting information

S1 Table PRISMA 2009 checklist. (DOC)

S2 Table Medline search strategy. (DOCX)

S3 Table Individual quality assessment of included studies. (DOCX)

S1 File Excluded studies. (DOCX)
