# Supplementary material for: Knowledge, beliefs, and concerns about bone health from a systematic review and metasynthesis of qualitative studies
Source: PLoS One. 2020 Jan 15;15(1):e0227765. doi: 10.1371/journal.pone.0227765 (PMC6961946; doi:10.1371/journal.pone.0227765)
Supplement: S2 Table — (DOCX) [file pone.0227765.s002.docx]

**S2 Table. Medline search strategy.**

| Line | **Terms** |
| --- | --- |
| 1 | exp OSTEOPOROSIS/ |
| 2 | OSTEOPOROTIC FRACTURES/ |
| 3 | BONE DENSITY/ |
| 4 | exp DENSITOMETRY/ |
| 5 | (bone health or BMD).ti,kw. |
| 6 | osteoporo*.ti,kw,jw. |
| 7 | ((bone* or skelet*) adj5 (densit* or fragil*)).ti,kw. |
| 8 | ((bone* or skelet*) adj5 (mass* or strength*)).ti,kw. |
| 9 | (bone* adj5 (lost or loss* or lose or losing)).ti,kw. |
| 10 | (osteopeni* or osteopaeni* or (fractur* adj5 risk*)).ti,kw. |
| 11 | (DEXA or DXA or FRAX).ti,kw. |
| 12 | ((((bone* or skelet*) adj5 (densit* or fragil* or mass* or strength* or lost or loss* or lose or losing)) or osteoporo* or osteopeni* or osteopaeni* or (fractur* adj5 risk*) or BMD or DEXA or DXA or FRAX) and (anxiet* or attitud* or awareness or belief* or believ* or concern* or disbelie* or distress* or knowledge or literacy or misunderstand* or perceive* or perception* or psycholog* or question** or uncertain* or understand* or worri* or educat* or self-efficacy or (health adj behav*) or perspective* or view or views or viewpoint* or "point of view*")).ti,kw. |
| 13 | ((((bone* or skelet*) adj5 (densit* or fragil* or mass* or strength* or lost or loss* or lose or losing)) or osteoporo* or osteopeni* or osteopaeni* or (fractur* adj5 risk*) or BMD or DEXA or DXA or FRAX) adj7 (anxiet* or attitud* or awareness or belief* or believ* or concern* or disbelie* or distress* or knowledge or literacy or misunderstand* or perceive* or perception* or psycholog* or question* or uncertain* or understand* or worri* or educat* or self-efficacy or (health adj behav*) or perspective* or view or views or viewpoint* or "point of view*")).ab. |
| 14 | or/1-13 |
| 15 | exp QUALITATIVE RESEARCH/ |
| 16 | FOCUS GROUPS/ |
| 17 | INTERVIEWS AS TOPIC/ |
| 18 | HEALTH IMPACT ASSESSMENT/ |
| 19 | or/15-18 [likely best study terms] |
| 20 | 14 and 19 |
| 21 | ((qualitative* or mixed method* or observation*) adj3 (study or studies or studied or studying or research or data or analy*)).ti,kw,ab. |
| 22 | (interview* or focus groups*).ti,kw,ab. |
| 23 | or/21-22 [additional study terms] |
| 24 | (14 and 23) not 20 |
| 25 | (anxiet* or attitud* or awareness or belief* or believ* or concern* or disbelie* or distress* or knowledge or literacy or misunderstand* or perceive* or perception* or psycholog* or question* or uncertain* or understand* or worri* or educat* or self-efficacy or (health adj behav*) or perspective* or view or views or viewpoint* or "point of view*").ti,kw. |
| 26 | 24 and 25 |
| 27 | (24 and 13) not 26 |
| 28 | exp *OSTEOPOROSIS/ or *OSTEOPOROTIC FRACTURES/ or *BONE DENSITY/ or exp *DENSITOMETRY/ or 5 |
| 29 | 27 and 28 |
| 30 | 20 or 26 or 29 [merging groups] |
| 31 | limit 30 to "all adult (19 plus years)" |
