# Supplementary material for: Knowledge, beliefs, and concerns about bone health from a systematic review and metasynthesis of qualitative studies
Source: PLoS One. 2020 Jan 15;15(1):e0227765. doi: 10.1371/journal.pone.0227765 (PMC6961946; doi:10.1371/journal.pone.0227765)
Supplement: S3 Table — (DOCX) [file pone.0227765.s003.docx]

**S3 Table. Quality assessment of each item for 24 of the 25 studies included in our metasynthesis.**

| Citation | Q1 | Q2 | Q3 | Q4 | Q5 | Q6 | Q7 | Q8 | Q9 | Q10 |
| --- | --- | --- | --- | --- | --- | --- | --- | --- | --- | --- |
| Backett-Milburn et al 2000 [8] | U | Y | Y | Y | Y | Y | Y | Y | Y | Y |
| Baheiraei et al 2006 [9] | U | U | U | U | Y | N | N | Y | Y | Y |
| Besser et al 2012 [10] | U | Y | Y | Y | Y | N | N | Y | Y | Y |
| Burgener et al 2005 [11] | U | U | U | U | U | N | N | Y | Y | Y |
| Hagy et al 2000 [13] | N | Y | Y | Y | Y | Y | N | Y | Y | Y |
| Hvas et al 2005 [32] | U | Y | Y | Y | Y | Y | Y | Y | Y | Y |
| Iversen et al 2011 [14] | U | Y | Y | Y | Y | N | N | Y | Y | Y |
| Jachna et al 2005 [15] | U | Y | Y | Y | Y | N | Y | Y | Y | Y |
| Lau et al 2008 [16] | Y | Y | Y | Y | Y | N | Y | Y | Y | Y |
| Mazor et al 2010 [17] | U | Y | Y | Y | Y | N | N | Y | Y | Y |
| Nielsen et al 2011 [19] | Y | Y | Y | Y | Y | N | N | Y | Y | Y |
| Nielsen et al 2013 [18] | Y | Y | Y | Y | Y | N | N | Y | Y | Y |
| Quantock et al 1997 [20] | U | Y | Y | U | Y | N | N | N | N | Y |
| Reventlow et al 2006 [21] | U | Y | Y | Y | Y | Y | Y | Y | Y | Y |
| Reventlow et al 2007 [22] | U | Y | Y | Y | Y | N | Y | Y | Y | Y |
| Richardson et al 2002 [23] | U | Y | Y | Y | Y | N | N | Y | Y | Y |
| Roberto et al 2001 [24] | U | Y | Y | Y | Y | N | N | Y | U | Y |
| Rothmann et al 2014 [25] | U | Y | Y | Y | Y | Y | Y | Y | Y | Y |
| Sale et al 2010 [26] | N/A | Y | Y | Y | Y | N | N | Y | Y | Y |
| Sale et al 2014 [27] | Y | Y | Y | Y | Y | Y | Y | Y | Y | Y |
| Skolbekken et al 2008 [28] | U | Y | Y | Y | Y | Y | Y | Y | Y | Y |
| Solimeo et al 2011 [29] | U | Y | Y | Y | Y | N | Y | Y | Y | Y |
| Unson et al 2001 [30] | U | Y | Y | Y | Y | N | N | Y | N | Y |
| Weston et al 2011 [31] | Y | Y | Y | Y | Y | Y | Y | Y | Y | Y |
| *% affirmative* | ***21*** | ***92*** | ***92*** | ***88*** | ***96*** | ***33*** | ***46*** | ***96*** | ***88*** | ***100*** |

Y, yes; N, no; U, unknown; N/A, not applicable.

**Q1,** Philosophical perspective and methodology are congruous

**Q2,** Methodology and the research question are congruous

**Q3,** Methodology and data collection methods are congruous

**Q4,** Methodology and analysis of data

**Q5,** Methodology and interpretation of results are congruous

**Q6,** Researcher culturally or theoretically located

**Q7,** Influence of the researcher addressed

**Q8,** Voices of participants represented

**Q9,** Study meets ethical criteria

**Q10,** Conclusions flow from data
