# Supplementary material for: Knowledge, beliefs, and concerns about bone health from a systematic review and metasynthesis of qualitative studies
Source: PLoS One. 2020 Jan 15;15(1):e0227765. doi: 10.1371/journal.pone.0227765 (PMC6961946; doi:10.1371/journal.pone.0227765)
Supplement: S1 File — (DOCX) [file pone.0227765.s004.docx]

**S1 File. Excluded studies.**

1. Beaton, DE, Sujic R, Beaton KM, Sale J, Elliot-Gibson V, Bogoch ER. Patient perceptions of the path to osteoporosis care following a fragility fracture. Qual Health Res. 2012;22(12):1647-1658.
2. Chapman KM, Chan MW. Focus groups: their role in developing calcium-related education materials. J Hum Nutr and Dietet. 1995;8(5):363-367.
3. Clarke LH, Liu-Ambrose T, Zyla J, McKay H, Khan K. ‘Being able to do the things that I want to do’: older women with osteoporosis define health, quality of life, and well-being. Act Adapt Aging. 2005;29(4):41-59.
4. Cott CA, Gignac MA. Independence and dependence for older adults with osteoarthritis or osteoporosis. Can J Aging. 1999;18(1):1-25.
5. Draper J, Roland M. Perimenopausal women’s views on taking hormone replacement therapy to prevent osteoporosis. BMJ. 1990;300(6727):786-788.
6. Drieling RL, Ma J, Thiyagarajan S, Stafford RS. An Internet-based osteoporotic fracture risk program: effect on knowledge, attitudes, and behaviors. J Womens Health. 2011;20(12):1895-1907.
7. Emmett CL, Redmond NM, Peters TJ, Clarke S, Shepstone L, Lenaghan E, Shaw AR. Acceptability of screening to prevent osteoporotic fractures: a qualitative study with older women. Family Practice. 2012;29(2):235-242.
8. Erol M. Melting bones: the social construction of postmenopausal osteoporosis in Turkey. Social Sci Med. 2011;73(10):1490-1497.
9. Feldstein AC, Schneider J, Smith DH, Vollmer WM, Rix M, Glauber H, Boardman DL, Herson M. Harnessing stakeholder perspectives to improve the care of osteoporosis after a fracture. Osteoporos Int. 2008;19(11):1527-1540.
10. French MR, Moore K, Vernace-Inserra F, Hawker GA. Factors that influence adherence to calcium recommendations. Can J Dietet Pract Res. 2005;66(1):25-29.
11. Gerend MA, Erchull MJ, Aiken LS, Maner JK. Reasons and risk: factors underlying women's perceptions of susceptibility to osteoporosis. Maturitas. 2006;55(3):227-237.
12. Giangregorio L, Dolovich L, Cranney A, Adili A, Debeer J, Papaioannou A, Thabane L, Adachi JD. Osteoporosis risk perceptions among patients who have sustained a fragility fracture. Patient Educ Couns. 2009;74(2):213-220.
13. Giangregorio L, Papaioannou A, Thabane L, DeBeer J, Cranney A, Dolovich L, Adili A, Adachi JD. Do patients perceive a link between a fragility fracture and osteoporosis? BMC Musculoskelet Disord. 2008;9:38.
14. Hammond GK, Chapman GE, Barr SI. Healthy midlife Canadian women: how bone health is considered in their food choice systems. J Hum Nutr Dietet. 2011; 24(1): 61-7.
15. Hansen C, Konradsen H, Abrahamsen B, Pedersen BD. Women’s experiences of their osteoporosis diagnosis at the time of diagnosis and 6 months later: a phenomenological hermeneutic study. Int J Qual Stud Health Well-being. 2014;9:22438.
16. Jensen AL, Harder I. The osteoporotic pain experience. Osteoporos Int. 2004;15(3):204-208.
17. Lee S, Ma GX, Juon HS, Martinez G, Hsu CE, Bawa J. Assessing the needs and guiding the future: findings from the health needs assessment in 13 Asian American communities of Maryland in the United States. J Immigr Minor Health. 2011;13(2):395-401.
18. Lespessailles E, Cotte FE, Roux C, Fardellone P, Mercier F, Gaudin AF. Prevalence and features of osteoporosis in the French general population: the Instant study. Joint, Bone, Spine: Revue du Rhumatisme. 2009;76(4):394-400.
19. Matsumoto D, Pun KK, Nakatani M, Kadowaki D, Weissman M, McCarter L, Fletcher D, Takeuchi S. Cultural differences in attitudes, values, and beliefs about osteoporosis in first and second generation Japanese-American women. Women Health. 1995;23(4):39-56.
20. McHorney CA, Schousboe JT, Cline RR, Weiss TW. The impact of osteoporosis medication beliefs and side-effect experiences on non-adherence to oral bisphosphonates. [Erratum appears in Curr Med Res Opin. 2008;24(3):707]. Curr Med Res Opin. 2007;23(12):3137-3152.
21. McKenna J, Ludwig AF. Osteoporotic Caucasian and South Asian women: a qualitative study of general practitioners' support. J R Soc Promot Health. 2008;128(5):263-270.
22. Ni Chroinin D, Glavin P, Power D. Awareness of osteoporosis, risk and protective factors and own diagnostic status: a cross-sectional study. Arch Osteoporos. 2013;8:117.
23. Nielsen D, Knold B, Ryg J, Nissen N, Huniche L, Brixen K. Education of patients in groups has a positive influence on conduct of everyday life of patients with osteoporosis - a qualitative study. Osteoporos Int. 2006;17:434-435.
24. Reventlow SD, Hvas L, Malterud K. Making the invisible body visible. Bone scans, osteoporosis and women’s bodily experiences. Soc Sci Med. 2006;62(11):2720-2731.
25. Reventlow S, Overgaard IS, Hvas L, Malterud K. Metaphorical mediation in women's perceptions of risk related to osteoporosis: a qualitative interview study. Health Risk Soc. 2008;10(2):103-115.
26. Ribeiro V, Blakeley J, Laryea M. Women’s knowledge and practices regarding the prevention and treatment of osteoporosis. Health Care Women Int. 2000;21(4):347-353.
27. Roberto KA, Gold DT, Yorgason JB. The influence of osteoporosis on the marital relationship of older couples. J Appl Gerontol. 2004;23(4):443-456.
28. Sale JE, Bogoch E, Hawker G, Gignac M, Beaton D, Jaglal S, Frankel L. Patient perceptions of provider barriers to post-fracture secondary prevention. Osteoporos Int. 2014;25(11):2581-2589.
29. Sale JE, Cameron C, Hawker G, Jaglal S, Funnell L, Jain R, Bogoch E. Strategies used by an osteoporosis patient group to navigate for bone health care after a fracture. Arch Orthop Trauma Surg. 2014;134(2):229-235.
30. Sale JE, Gignac MA, Frankel L, Hawker G, Beaton D, Elliot-Gibson V, Bogoch E. Patients reject the concept of fragility fracture—a new understanding based on fracture patients’ communication. Osteoporos Int. 2012;23(12):2829-2834.
31. Sale JE, Hawker G, Cameron C, Bogoch E, Jain R, Beaton D, Jaglal S, Funnell L. Perceived messages about bone health after a fracture are not consistent across healthcare providers. [Erratum appears in Rheumatol Int. 2015;35(1):105; PMID: 25059574]. Rheumatol Int. 2015;35(1):97-103.
32. Salter C, McDaid L, Bhattacharya D, Holland R, Marshall T, Howe A. Abandoned acid? Understanding adherence to bisphosphonate medications for the prevention of osteoporosis among older women: a qualitative longitudinal study. PLoS ONE. 2014;9(1):e83552.
33. Sandison R, Gray M, Reid DM. Lifestyle factors for promoting bone health in older women. J Adv Nurs. 2004;45(6):603-610.
34. Saw SM, Hong CY, Lee J, Wong ML, Chan MF, Cheng A, Leong KH. Awareness and health beliefs of women towards osteoporosis. Osteoporos Int. 2003;14(7):595-601.
35. Sharts-Hopko NC, Smeltzer S. Perceptions of women with multiple sclerosis about osteoporosis follow-up. J Neurosci Nurs. 2004;36(4):189-194, 199.
36. Sharts-Hopko NC, Sullivan MP. Beliefs, perceptions, and practices related to osteoporosis risk reduction among women with multiple sclerosis. Rehab Nurs J. 2002;27(6):232-236.
37. Shulha JA, Sviggum CB, O’Meara JG, Berg ML. Assessment of the presence and quality of osteoporosis prevention education among at-risk internal medicine patients. Consultant Pharmacist. 2014;29(1):39-46.
38. Sriring P, Krass I, Kanjanarach T. Calcium consumption for osteoporosis prevention: knowledge, attitudes and behavior in the northeastern region, Thailand. J Med Assoc Thailand. 2014;97(2): 232-240.
39. Stoffman N, Schwartz B, Austin SB, Grace E, Gordon CM. Influence of bone density results on adolescents with anorexia nervosa. Int J Eating Disord. 2005;37(3):250-255.
40. Unson CG, Siccion E, Gaztambide J, Gaztambide S, Mahoney Trella P, Prestwood K. Nonadherence and osteoporosis treatment preferences of older women: a qualitative study. J Womens Health. 2003;12(10):1037-1045.
41. Werner P, Olchovsky D, Erlich-Gelaki H, Vered I. First-degree relatives of persons suffering from osteoporosis: beliefs, knowledge, and health-related behavior. Osteoporos Int. 2003;14(4):306-311.
42. Wilkins S. Women with osteoporosis: strategies for managing aging and chronic illness. J Women Aging. 2001;13(3):59-77.
43. Winzenberg T, Hansen E, Jones G. How do women change osteoporosis-preventive behaviours in their children? Eur J Clin Nutr. 2008;62(3):379-385.
